# Systematic review and meta-analyses of randomized controlled trials examining tinnitus management

**DOI:** 10.1002/lary.21825

**Published:** 2011-07

**Authors:** Derek J Hoare, Victoria L Kowalkowski, Sujin Kang, Deborah A Hall

**Affiliations:** 1 National Institute for Health Research National Biomedical Research Unit in HearingNottingham; 2 School of Clinical Sciences, The University of NottinghamNottingham; 3 Division of Psychology, School of Social Sciences, Nottingham Trent UniversityNottingham, United Kingdom

**Keywords:** Tinnitus, Good Practice Guidelines, UK Department of Health, cognitive behavioral therapy, tinnitus retraining therapy, randomized controlled trial, Level of Evidence: 1a.

## Abstract

**Objectives/Hypothesis:**

To evaluate the existing level of evidence for tinnitus management strategies identified in the UK Department of Health's Good Practice Guideline.

**Study Design:**

Systematic review of peer-reviewed literature and meta-analyses.

**Methods:**

Searches were conducted in PubMed, Cambridge Scientific Abstracts, Web of Science, and EMBASE (earliest to August 2010), supplemented by hand searches in October 2010. Only randomized controlled trials that used validated questionnaire measures of symptoms (i.e., measures of tinnitus distress, anxiety, depression) were included.

**Results:**

Twenty-eight randomized controlled trials met our inclusion criteria, most of which provide moderate levels of evidence for the effects they reported. Levels of evidence were generally limited by the lack of blinding, lack of power calculations, and incomplete data reporting in these studies. Only studies examining cognitive behavioral therapy were numerous and similar enough to perform meta-analysis, from which the efficacy of cognitive behavioral therapy (moderate effect size) appears to be reasonably established. Antidepressants were the only drug class to show any evidence of potential benefit.

**Conclusions:**

The efficacy of most interventions for tinnitus benefit remains to be demonstrated conclusively. In particular, high-level assessment of the benefit derived from those interventions most commonly used in practice, namely hearing aids, maskers, and tinnitus retraining therapy needs to be performed.

## INTRODUCTION

The “phantom” auditory experience of tinnitus affects 10% to 15% of people in the United Kingdom.^1^ It is most clearly associated with noise exposure and ageing and can present with comorbid sleep disturbance, hearing difficulty, social withdrawal, and negative emotional reactions such as anxiety and depression.^2^, ^3^ Although a majority of tinnitus patients are male and present with some form of hearing loss and higher-frequency steeply sloping hearing loss in particular, there is no typical characteristic history or level of distress of the help-seeking tinnitus patient.^4^

In the United Kingdom, the Department of Health issued a Good Practice Guide (GPG) for the commissioning of tinnitus services and for the clinical assessment and management of tinnitus patients.^5^ It recommends that tinnitus severity is assessed using either the Tinnitus Handicap Inventory (THI)^6^ or the Tinnitus Questionnaire.^7^ It also recommends that psychological comorbidity is assessed using the Beck Depression Inventory (BDI) and Beck Anxiety Inventory^8^ or the Hospital Anxiety and Depression Scale (HADS).^9^ However, questionnaire use in clinical practice is limited.^10^

In terms of management, therapeutic targets include the sound itself (i.e., interrupt the neural signal generating the sound) or the associated symptoms of distress, anxiety, or depression that can accompany tinnitus. The GPG states that tinnitus patients will be given, as appropriate, information/education, hearing aids, counseling and psychological support, relaxation therapy, cognitive behavioral therapy (CBT) with the requisite professional supervision, sleep management (including supervised CBT), sound enrichment therapy, and tinnitus retraining therapy (TRT). The use of antidepressants, anxiolytics, and night sedation is also advocated. However, the GPG lacks an evidence base to support these recommendations and is in part based on anecdotal evidence and expert opinion.

This review asked the question, how much high-level evidence exists for the efficacy of the GPG-suggested tinnitus management strategies, and was restricted to randomized controlled trials (RCTs), the gold standard for evaluating therapeutic interventions.^11^, ^12^ A systematic search was used to identify RCTs that examined tinnitus management strategies and reported validated measures of tinnitus intrusiveness, anxiety, or depression, therefore providing comparable measures of change in patient symptoms. Where possible, meta-analyses were conducted. The outcome of this review provides high-level evidence that supports some, but not all, GPG recommended strategies for tinnitus management in the United Kingdom, and also highlights where further RCTs would be of immediate benefit.

## MATERIALS AND METHODS

The PRISMA Statement outlines the essential components for transparent reporting of a systematic review^13^ and guided our reporting.

### Systematic Search Strategy and Study Selection

Studies were selected and screened according to the research question and PICOS criteria. PICOS were: participants, adult humans with tinnitus; intervention, tinnitus management strategies proposed by the Department of Health (GPG); comparisons, a no-treatment group or suitable second treatment group; outcomes, validated questionnaire measure of tinnitus intrusiveness, anxiety, depression; and study design, randomized controlled trials only. Quasi- and pseudo-randomizations were excluded from this review.

**Fig 1:**
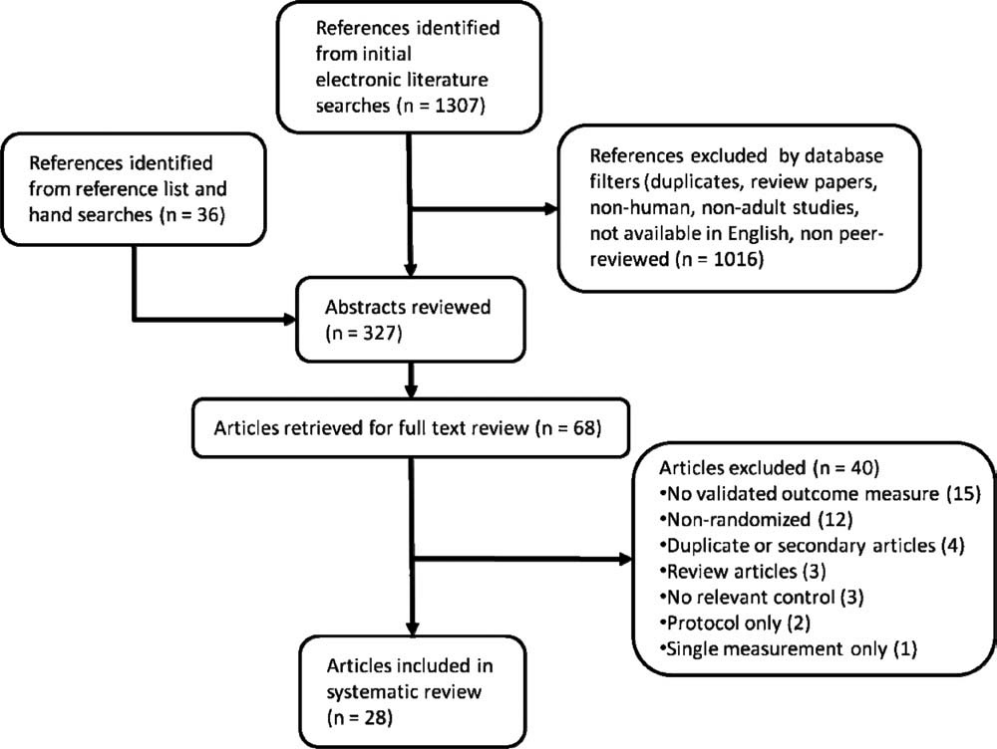
Summary of the systematic literature search.

Initial database searches were conducted in August 2010. PubMed, Cambridge Scientific Abstracts (including Medline, Toxbase, Biological Sciences), Web of Science, and EMBASE were searched using the search terms: 1) tinnitus AND 2) random AND/OR randomized AND/OR randomised AND 3) information AND/OR education OR hearing AND aid(s) OR counselling OR counseling AND/OR psychological (support) OR relaxation (therapy) OR cognitive AND behavioural OR behavioral AND therapy AND/OR CBT OR sleep AND management OR sound AND enrichment (therapy) OR retraining AND therapy AND/OR TRT OR antidepressant OR depression or anxiolytic OR anxiety OR night AND/OR nocturnal AND sedation. Search results were screened to remove duplicate, non-peer reviewed, and review articles. Two authors (D.J.H., V.L.K.) screened abstracts according to the research question and PICOS criteria. All titles selected by either author were retrieved to screen their full text for final agreement and inclusion. The initial search was complimented with further hand searches of key journals, Cochrane reviews, and the reference lists of all included studies. The stages and reasons for exclusions are presented in Figure 1.

### Data Extraction

Extracted data comprised participant numbers, baseline tinnitus severity, age range, the intervention and control, validated questionnaire measures used, follow-up conducted, the study design, and study findings. For all studies, data was extracted independently by two authors (D.J.H., V.L.K.). Any differences in reporting were reconciled by jointly revisiting the relevant publication.

### Quality Assessment

Study quality was based on a subset of extracted data. The quality assessment tool was based on one reported previously.^14^ For each criterion, a score of 2 was applied if the study met the criterion to a high standard, 1 if it partly met the criterion, or a score of 0 if it was flawed or if the relevant information was not stated. Two authors scored studies independently before agreeing on a final score for each criterion and each study. The potential overall quality score varied from 0 to 16, giving grades of evidence from very low (0–4), low (5–8), moderate (9–12) to high (13–16). Qualitative descriptors for these grades are given in Oxman et al.^11^ Essentially, the lower the level of evidence, the more likely further studies are to impact on our confidence in the estimate of effect and are likely to change that estimate. Only high-level evidence studies provide conclusive evidence, after which additional research is unlikely to change the estimate of effect.

### Data Synthesis and Meta-Analyses

Where studies were sufficiently similar, a meta-analysis was performed. Similarity is defined here as the same intervention and outcome measure, and low heterogeneity. The test for heterogeneity is detailed below. Where studies were not sufficiently similar to conduct a meta-analysis, data were synthesized using a narrative approach. All analyses were performed in *R* using libraries rmeta, epiR, and meta.

### Effect Size Calculation for Individual Studies

For each study, Cohen's *d* effect size was calculated for the primary treatment intervention and primary measure of change compared to an independent control given by the formula below. An intention-to-treat analysis was used, and follow-up assessments were not included in this analysis. Limited by the available data, standard deviation (SD) of preintervention and precontrol (baseline) scores were used as the group-specific standard deviations of mean change scores within groups.


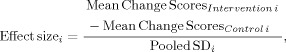


where

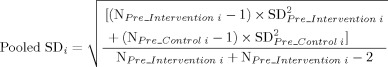


and SDs and Ns are the group-specific standard deviations and sample sizes for study *i*.

### Test for Heterogeneity Across Studies

Heterogeneity *(χ*^*2*^*)* of aggregated effect sizes were calculated using Cochran's *Q* statistic (significance level =.05) and *I*^2^ index (percentages of around 25% indicates low heterogeneity and 0 indicates that sampling error only accounts for all the variability in effect size estimates within studies). Results were checked for significance with K-1 degrees of freedom, where K = number of studies.

### Meta-Analysis: Summary Effect Size and Confidence Intervals

Fixed-effects meta-analyses of questionnaire measures were performed, using the standardized mean difference method. In addition, weighted meta-analysis with fixed-effects standard errors was performed to get a summary effect size (and its 95% confidence interval [CI]) for each type of intervention as appropriate. Weighting used the inverse variance procedure, where studies with a large standard error, typically with small sample sizes, were given less weight.^15^ The statistical significance of the summary effect size was evaluated using the Z statistic (Z_*i*_ = T_*i*_/se(T_*i*_)where T_*i*_ is the effect measure from study *i*).^15^

### Estimating Publication Bias Effects

To search for evidence of publication bias and evaluate the influence of individual studies on the outcome, funnel plots were constructed. The estimate of effect (observed outcome) from each study was plotted against its standard error. If there is no publication bias, the data-points appear symmetrical around the mean effect. An asymmetrical plot, however, implies that studies giving significant results were more likely to be published than studies giving nonsignificant results.

The “trim and fill” method^16^ was applied to calculate the number of studies that would be required to return the plot to “symmetry,” and therefore remove publication bias. This method estimates summary effect size based on the assumption that all such studies had been published.

## RESULTS

All studies included in this review were RCTs of tinnitus management interventions mentioned in the GPG. All participant groups were representative of a clinically relevant population, and without exception all studies reported one or more outcomes based on validated questionnaire measures of tinnitus, anxiety, or depression. Unless otherwise stated, studies included adults of all ages, and baseline scores for outcome measures did not differ significantly between groups. Throughout, statistical significance refers to a reliable numerical difference between group means. Clinical significance refers to the specific questionnaire score change that implies a functional improvement or worsening of the condition in an individual patient.

A descriptive summary table of all 28 studies is provided in the online supplemental information accompanying this article. Table I shows the quality assessment scores for each study.

**Table I:** Assessment of Eight Quality Criteria, Overall Quality Rating, and Effect Size for Each of the 28 Studies

Reference	Study Design*	Blinding†	Outcome Measures‡	Compliance§	Controls||	Similarity Between Groups¶	Potential Bias#	External Validity**	Study Quality	Cohen's *d* Effect Size (Variable)
Information/education										
Henry et al. (2007)^17^	2	0	2	1	2	2	2	1	Moderate	—
Kaldo et al. (2007)^22^	2	0	2	2	1	2	2	1	Moderate	0.52 (TRQ)
Malouff et al. (2010)^23^	1	0	2	1	1	1	2	1	Moderate	0.25 (TRQ)
Relaxation therapy										
Biesinger et al. (2010)^25^	1	0	2	2	1	1	1	1	Moderate	0.43 (TBF-12)
Ireland et al. (1985)^27^	2	0	1	0	1	1	1	1	Low	0.05 (BDI)
Weise et al. (2008)^29^	2	0	2	1	1	2	2	2	Moderate	1.63 (TQ)
Cognitive behavioral therapy										
Abbott et al. (2009)^31^	1	0	2	2	2	1	1	0	Moderate	0.21 (TRQ)
Andersson et al. (2002)^30^	2	0	2	2	1	1	2	2	Moderate	0.45 (TRQ)
Andersson et al. (2005)^34^	2	0	2	1	1	2	2	1	Moderate	0.65 (TRQ)
Henry and Wilson (1996)^19^††	2	0	2	1	2	1	1	1	Moderate	0.37 (TRQ)
Henry and Wilson (1998)^36^	1	0	2	1	2	0	1	1	Low	0.6 (TRQ)
Kaldo et al. (2008)^32^	2	0	2	1	1	2	1	1	Moderate	−0.18 (TRQ)
Kröner-Herwig et al. (1995)^37^	2	0	2	1	1	2	1	1	Moderate	—
Kröner-Herwig et al. (2003)^39^‡‡	2	0	2	1	2	1	2	1	Moderate	0.67 (TQ)
Rief et al. (2005)^42^	2	0	2	1	1	2	1	2	Moderate	0.58 (TQ)
Robinson et al. (2008)^38^	2	1	2	2	1	1	2	2	High	—
Zachriat and Kröner-Herwig (2004)^41^§§	1	0	2	1	2	2	1	1	Moderate	0.66 (TQ)
Sound enrichment										
Davis et al. (2008)^43^	0	0	2	1	2	1	0	1	Low	—
Herraiz et al. (2010)^45^	2	0	2	2	1	2	2	1	Moderate	0.44 (THI)
Stephens and Corcoran (1985)^24^	1	0	1	0	2	1	2	1	Low	—
Tinnitus retraining therapy										
Caffier et al. (2006)^47^	2	0	2	0	1	1	1	1	Low	—
Seydel et al. (2010)^48^	2	0	2	0	1	1	1	1	Low	—
Antidepressants										
Robinson et al. (2005)^49^	2	2	2	2	2	2	1	2	High	0.09 (THQ)
Sullivan et al. (1993)^50^	1	2	2	1	2	1	2	1	Moderate	0.88 (HAM-D)
Zöger et al. (2006)^52^	1	2	2	2	2	2	1	2	High	0.35 (HAM-D)
Anxiolytics										
Jalali et al. (2009)^56^	2	2	2	1	1	1	2	2	High	0.07 (THI)
Night sedation										
Neri et al. (2009)^57^	2	0	2	2	1	1	1	1	Moderate	—
Rosenberg et al. (1998)^58^	2	2	2	2	2	2	1	1	High	—

* Study design: fully randomized (score 2) or stratified (score 1).

† Blinding of participants (score 1) and investigators (score 1).

‡ Validated questionnaire measures (score 2 if appropriate, 1 if weak).

§ Compliance measures (score 1) and reporting (score 1).

|| Quality of control condition (score 2 if well-controlled intervention, score 1 for waiting-list control).

¶ Similarity of groups: baseline tinnitus severity (score 2 if no significant differences, 1 if minor differences or under-reported, 0 if groups not matched).

# Bias related to funding (score 2 if stated and no evidence of bias, 1 if academic with no statement of funding source).

** External validity representative a clinical population (score 1); power calculation was conducted (score 1).

†† Also examined education.

‡‡ Also examined education and relaxation.

§§ Also examined TRT.

TRQ = Tinnitus Reaction Questionnaire, TBF-12 = Tinnitus Handicap Inventory-12, BDI = Beck Depression Inventory, TQ = Tinnitus Questionnaire (Goebel-Hiller); THI = Tinnitus Handicap Inventory, THQ = Tinnitus Handicap Questionnaire, HAM-D = Hamilton Depression Scale.

### Information/Education (Directive Counseling)

Three RCTs were identified that assessed the efficacy of education/information giving. They provide moderate levels of evidence and report small or moderate effect sizes (Table I). Henry et al.^17^ found a statistically significant reduction in Tinnitus Severity Index^18^ score at 6-month follow-up in a group receiving a tinnitus education program delivered by audiologists, compared to a change in members of a self-help group. Differences at all other time points were not significant. In a study of CBT discussed later, Henry and Wilson^19^ examined the efficacy of group education delivered by a clinical psychologist versus a waiting-list control. In this study they found no significant changes in the BDI, Tinnitus Handicap Questionnaire (THQ),^20^ or Tinnitus Reaction Questionnaire (TRQ).^21^

Two further studies looked at the efficacy of a self-help book with or without telephone contact with a therapist, compared to a waiting list control.^22^, ^23^ Kaldo et al.^22^ reported a significant reduction in TRQ score in their intervention group compared to controls. Whether benefit was retained is unclear, as 15 participants tried additional interventions between the end of intervention and follow-up. In terms of clinically significant changes in TRQ (i.e., a 50% reduction),^21^ 32% of the intervention group and 5% of the control group improved. In the second study, Malouff et al.^23^ used the same self-help book but without therapist contact by phone. Despite a high attrition rate (35%), they also reported a statistically significant improvement in mean TRQ score. Twenty-five percent of the intervention group had a clinically significant reduction in TRQ score versus 14% of controls. Because Kaldo et al.^22^ used a self-help book with weekly therapist phone calls and Malouff et al.^23^ used only the self-help book, meta-analysis could not be performed.

In summary, information/education delivered in the form of a book or therapist-led educational sessions had significant effects over no intervention or undirected self-help. Furthermore, providing a self-help book and therapist contact appears to have greater benefit than providing a self-help book alone. Education alone is sufficient for some patients because it also makes them aware of other potential management interventions (i.e., some individuals may use that information to explore other strategies, as was observed by Kaldo et al.^22^).

### Hearing Aids

No RCTs investigated hearing-aid use as a primary intervention for tinnitus and obtained a validated outcome measure. Stephens and Corcoran^24^ did include a subgroup of individuals who received hearing aids (discussed later under sound enrichment). Sometimes hearing-aid provision forms part of TRT, and therefore these studies are discussed later.

### Counseling and Psychological Support

No specific RCTs involved counseling or psychological support. When counseling was given, it was more appropriate to consider it as information/education, relaxation therapy, CBT, or TRT.

### Relaxation Therapy

Three RCTs assessed the efficacy of relaxation therapies, providing low to moderate levels of evidence, of small to large effect sizes (Table I). Biesinger et al.^25^ examined the efficacy of Qigong, a mindful exercise and active relaxation technique. They reported high compliance and a significant mean improvement in Tinnitus Beeinträchtigungs Fragebogen Questionnaire^26^ score in the intervention group versus waiting-list control. However, in this study the control group reported a significantly higher baseline tinnitus severity, limiting the interpretative weight of their finding. In a second study, Ireland et al.^27^ compared group (progressive muscle) relaxation training to a waiting-list control. They found no significant differences in change on BDI or State-Trait Anxiety Inventory scores^28^ between groups. They did, however, note greater improvement in three participants who said their tinnitus was stress related. This study was rated as low-level evidence and did not report a validated measure of tinnitus distress. The third study of relaxation therapy from Weise et al.^29^ used electromyography biofeedback with a waiting-list control. They found significant improvements in both Tinnitus Questionnaire (Goebel-Hiller) (TQ) and BDI scores in their intervention group. The methodologies used in the three studies of relaxation training were not sufficiently similar to perform meta-analysis.

In summary, there is evidence for the efficacy of relaxation therapy for tinnitus intrusiveness, mixed evidence of its efficacy for depressive symptoms, and no evidence for an effect on anxiety.

### Cognitive Behavioral Therapy

Eleven RCTs examined CBT for tinnitus, the majority of which provided moderate levels of evidence (Table I). Three studies examined the efficacy of CBT delivered via the internet.^30–32^ Andersson et al.^30^ examined the efficacy of internet-based CBT but compared to a waiting-list control. Attrition was 51% in this study (leaving fewer participants than their calculated sample size). They did, however, report significant statistical improvements in TRQ score in the CBT group compared to controls. Thirty-one percent had a clinically significant improvement. Andersson et al.^30^ also reported statistically significant improvements in both anxiety and depression. In a second study from the same research group, Abbott et al.^31^ compared internet-based CBT to internet-based education without CBT elements. They reported 75% (24 of 32) attrition for the CBT intervention and 21% in controls. Participants reported that the intervention was not engaging enough. The authors also suggested that this intervention may be too much of a commitment and therefore inappropriate for those with low baseline tinnitus distress levels. Unsurprisingly, no significant effects on TRQ or Depression, Anxiety and Stress Scale^33^ scores were identified. In a third study, Kaldo et al.^32^ compared internet-delivered CBT with therapist-delivered group CBT. This study found no significant differences in THI, TRQ, or HADS score changes between groups (i.e., one mode of delivery was not superior to the other). In this study, 38% of participants failed to complete the internet CBT compared to 24% who received the group CBT intervention. Internet CBT was deemed less credible by participants.

Four studies examined the efficacy of CBT delivered as group sessions by either clinical psychologists or a psychiatrist, compared to waiting-list controls. Andersson et al.^34^ made this comparison in elderly tinnitus patients. They found a statistically significant improvement in TRQ score for their CBT group compared to controls. There were no significant improvements in HADS or Anxiety Severity Index (ASI)^35^ scores. In fact, the ASI score increased significantly in their control group. In terms of clinical significance, 42% of CBT participants showed clinical improvement in TRQ, but this reduced to 25% at 3-month follow-up. Henry and Wilson^36^ also reported statistically significant improvement in TRQ score in their CBT group compared to controls. They found no changes in either BDI or THQ scores. Kröner-Herwig et al.^37^ reported a significant improvement in TQ score. In Robinson et al.,^38^ attrition was 32% in the CBT group (participants reported that they were not distressed enough or that participation was too demanding on time). Noncompleters had experienced tinnitus significantly longer than those who completed the intervention, suggesting that CBT is less indicated in very chronic cases. A power calculation was conducted for this study, but again, because of high attrition, the sample size estimate was not met. Furthermore, as is unforeseeable with full randomization, the intervention group had a significantly higher baseline TRQ than controls, making the groups less comparable. A significant effect was also reported for changes in BDI score in the CBT group compared to controls.

Three studies compared the efficacy of psychologist-delivered group CBT to education or relaxation training. Henry and Wilson^36^ examined group CBT versus education alone, as well as waiting-list controls, and reported a significant reduction in TRQ score in the CBT. However, the difference between groups was not significant at 12-month follow-up. THQ score also reduced significantly in their CBT group versus controls. There was no significant effect on BDI scores. Kröner-Herwig et al.^39^ compared group CBT to a minimal contact education or relaxation intervention and a waiting-list control. The group who received CBT were significantly more improved on TQ score than all other groups. Changes in the General Depression Scale (ADS)^40^ were not significant, which the author attributes to “floor effects.” In this study, the groups who received either the education or relaxation intervention did not show significant improvement in TQ scores compared to controls. In the third study, Zachriat and Kröner-Herwig^41^ compared CBT, TRT, and an education-only intervention. TQ scores were significantly improved in the CBT group compared to controls. The difference between groups remained significant 6 months following intervention but was not significant at a subsequent follow-up.

The final study of CBT reviewed here, from Rief et al.,^42^ examined the efficacy of one-to-one CBT compared to waiting-list controls. They found a significant improvement in TQ score for the CBT group compared to controls. The benefit was maintained after 6 months.

**Fig 2:**
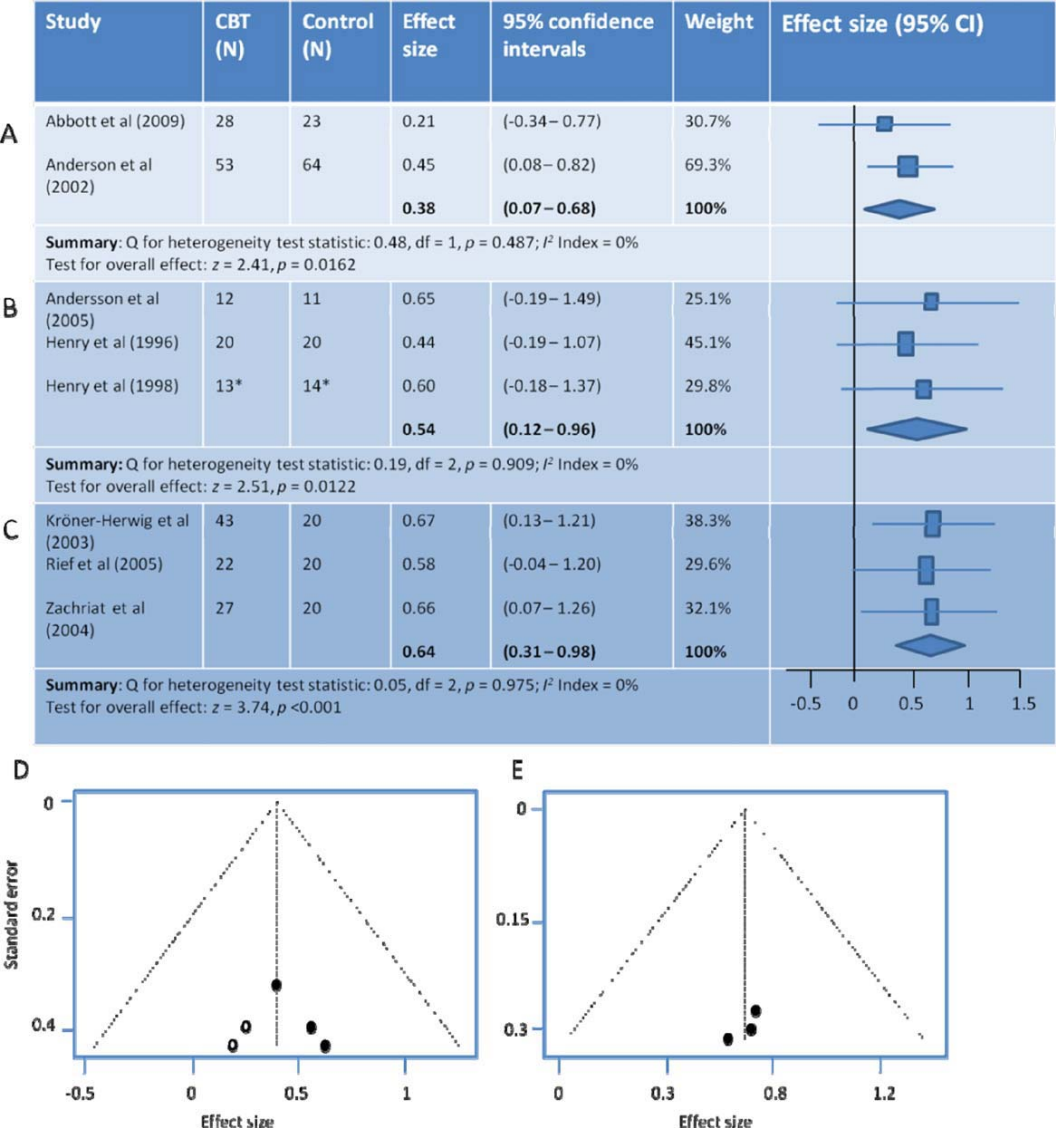
Meta-analyses of cognitive behavioral therapy (CBT) interventions. Pooled effect sizes and 95% confidence intervals (CI) are given in bold and as diamond shapes in the forest plot (right). (A) Comparison of internet-CBT versus control using Tinnitus Reaction Questionnaire (TRQ) total scores preintervention and postintervention. (B) Comparison of therapist-delivered CBT versus control TRQ total scores preintervention and postintervention. (C) Comparison of therapist-delivered CBT versus control Tinnitus Questionnaire (TQ) total scores preintervention and postintervention. (D) Funnel plot shows estimated effect against standard error (SE) for the therapist delivered CBT study using TRQ (filled circles). There is considerable asymmetry around mean effect size (evidence of publication bias). Open circles show calculated position of results from studies that would be required to bring the plot back to symmetry. (E) Funnel plot shows estimated effect against SE for the therapist delivered CBT studies using TQ. There is good symmetry and therefore no evidence of publication bias. *Sample sizes were assumed. [Color figure can be viewed in the online issue, which is available at wileyonlinelibrary.com.]

Effect sizes were calculated for each study of CBT (Table I). Meta-analysis was performed to determine overall effect sizes comparing the pre-change versus post-change, and 95% CIs for CBT when delivered via the internet (Fig. 2A) or by a therapist (Fig. 2B, 2C). Because Kaldo et al.^32^ used standard group-based CBT as a control group and Robinson et al.^38^ did not provide postintervention data, they were excluded from meta-analyses.

There was no significant heterogeneity or degree of inconsistency between any of the studies grouped for meta-analysis (*P* = not significant, *I*^2^ index = 0% in all cases).

The overall effect size for internet-delivered CBT (Fig. 2A) was 0.38 (small effect) with a 95% CI of 0.07–0.68 (*z* = 2.41 and *P* =.016) (i.e., patients undergoing internet-delivered CBT reported significant improvement compared to controls).

The overall effect size for therapist-delivered CBT when measured using THQ scores (Fig. 2B) was 0.54 (medium effect) with a 95% CI of 0.12-0.96 (*z* = 2.51 and *P* =.012) (i.e., the patients undergoing CBT improved). The funnel plot (Fig. 2D) for these studies suggests that two additional studies would need to be added to bring the funnel plot back to symmetry. If these two studies were added then the estimated treatment effect (fixed-effect model) would drop from 0.54 to 0.44.

A final meta-analysis was performed for therapist-delivered CBT when measured using the TQ (Fig. 2C). The overall effect size was 0.64 (medium effect) with a 95% CI of 0.31 to 0.98 (*z* = 3.74 and *P* < 0.001). Again therefore, patients undergoing therapist-delivered CBT reported significant improvement compared to controls. The funnel plot for the therapist-delivered CBT with TQ (Fig. 2E) shows considerable symmetry (i.e., there is no evidence of bias, and therefore we can be confident of this effect).

In summary, 10 RCTs compared CBT to a non-CBT control, of which nine reported significant improvements in tinnitus intrusiveness. Of the seven studies that measured depression, only two found improvements, and of the three studies that measured anxiety, just one reported improvement. CBT is therefore specifically associated with reductions in self-reported tinnitus severity with little evidence to suggest it has any benefit for tinnitus-related anxiety or depression. This effect appears to be independent of whether CBT is compared to an educational or waiting-list control, suggesting that either measure adequately controls for placebo effects in these studies.

The effect size for therapist-delivered CBT is larger than that for internet CBT, although the one study that assessed the relative efficacy of these two methods (Kaldo et al.)^32^ did not find any significant differences in their outcome measures (HADS, THI, or TRQ). Of note in this study, however, is that email contact, part of the internet-delivered CBT, was tailored to perceived need, whereby considerably more email contact was maintained with some participants than might have occurred in other studies. Although internet-delivered CBT might be accessible and cost effective, it had the highest attrition of all interventions in this review.

Subanalyses conducted in studies of CBT do not suggest that the intervention is less indicated for patients with milder tinnitus or longer-term tinnitus, and does not appear to be effective alone for depressive or anxiety symptoms.

Effective CBT interventions were either delivered as a software package, or face-to-face by a clinical psychologist or psychiatrist. Crucially, no RCT was identified where CBT was delivered by an audiologist or hearing therapist. Given the limited availability of clinical psychology support in the United Kingdom,^10^ it is important that the efficacy of audiologist-delivered CBT, as advocated by the GPG, is assessed with an RCT. Furthermore, individual studies report delivering CBT for between 7 and 22 hours in total, over a period of 6 to 15 weeks. The issues of patient and clinician burden, and of cost benefit, are therefore also important considerations.

### Sleep Management

No RCTs specifically examined sleep management practices for tinnitus patients, beyond those for CBT or night sedation.

### Sound Enrichment Therapy

Three RCTs assessed the efficacy of different forms of sound enrichment. These studies provide low to moderate levels of evidence, and where calculable, indicate a small effect size (Table I). Davis et al.^43^ examined the efficacy of Neuromonics (customized spectrally modified music) compared to counseling with or without a noise generator. The original study design included two groups with different modules of Neuromonics intervention, but participants self-adjusted the prescribed treatment for what they felt worked best, such that that the intervention was no longer different between groups and their data was pooled. Davis et al.^43^ reported clinically significant changes in TRQ at 6 months for 86% of Neuromonics participants; 47% of participants received noise generator and counseling, and just 23% received counseling alone. Although this is encouraging, study quality was rated low because of inadequate randomization, a lack of blinding, and under-reporting of study detail, therefore there is significant uncertainty of the estimated effect. In an earlier study, Stephens and Corcoran^24^ examined the efficacy of maskers, hearing aids or combination devices, tailored to individual hearing loss, compared to limited counseling without a sound device. There were no improvements in this study. In fact, one group using maskers reported a significant increase in anxiety (Crown Crisp Experiential Index)^44^ compared to controls. This study also provided only low-level evidence of the reported effect, but given the lack of evidence to suggest otherwise, contraindicates masking devices for tinnitus patients presenting with anxiety.

An active form of sound enrichment was used by Herraiz et al.^45^ Participants undertook frequency discrimination training, after which there was a reduction in THI score in their intervention groups compared to waiting-list controls. Although this was statistically significant, it was not clinically significant. This finding may be in part limited by their eligibility criteria, which excluded those with more severe tinnitus.

Sound enrichment interventions and outcome measures were not sufficiently similar for us to perform meta-analysis. It is surprising that so little evidence exists for the efficacy of sound enrichment given that, in the United Kingdom at least, prescription of hearing aids and maskers for tinnitus is commonplace.^10^ In particular, it is crucial to establish the efficacy of hearing aids for patients with mild hearing loss, who may or may not be offered a hearing aid depending on the clinicians' own personal belief about efficacy. This is quite a widespread practice in the United Kingdom.^10^

### Tinnitus Retraining Therapy

The aim of TRT is to promote habituation to tinnitus through a combination of sound enrichment and directive counseling.^46^ In addition to the study reported earlier (Zachriat and Kröner-Herwig),^41^ two further RCTs of TRT were identified, both of which provide low-level evidence. Caffier et al.^47^ compared TRT to a waiting-list control, finding a significant reduction in TQ score in their TRT group versus controls at 12 months. Interpreting efficacy is difficult. TRT was not delivered according to a single protocol but was highly individual between participants, as was the use of prescribed devices, and additional treatment for anxiety or depression. Authors indicate that there was more improvement in participants undertaking (as opposed to rejecting) additional treatment for anxiety or depression if it was indicated. Seydel et al.^48^ compared TRT (modified to include psychosomatic treatment, cognitive-behavioral elements, and relaxation treatment) with a waiting-list control. After 3 months of treatment, mean TQ score was significantly reduced in the TRT group compared to controls. ADS scores, indicating anxiety and depression, also reduced significantly in the TRT group compared to controls. The authors noted greater improvement in individuals with a higher baseline depression score. Follow-up was not well controlled, but the TQ score of the TRT group remained low after 1 year, whereas ADS score returned to baseline after 6 months. As in Caffier et al.^47^ treatment was tailored to individual need.

The efficacy of TRT may depend on its adaptability as an intervention rather than as a fixed, protocol-driven intervention. The two studies of TRT were not sufficiently similar to perform meta-analysis.

### Antidepressants

Three RCTs examining the efficacy of antidepressants were identified, providing moderate-to-high level evidence of negligible to large-effect sizes (Table I). Robinson et al.^49^ compared paroxetine (up to 50 mg daily, as tolerated) to placebo. They found no significant changes in tinnitus intrusiveness, anxiety, or depression. Furthermore, they noted several significant adverse events: sexual dysfunction, excessive drowsiness, and dry mouth. Sullivan et al.^50^ compared nortriptyline (up to 100 mg at night, as tolerated) to placebo (lactose). They reported a significant reduction in Hamilton Depression Rating Scale,^51^ score for the intervention group compared to controls. Sub-analysis suggested that those reporting greatest benefit were women and individuals reporting insomnia. Zöger et al.^52^ compared sertraline to placebo. They reported significant improvements on the Tinnitus Severity Questionnaire^53^ and the Hamilton Anxiety Scale (HAM-A)^54^ for the group receiving sertraline compared to placebo. This study is slightly complicated, as nine participants were given oxazepam in the first 2 weeks to alleviate expected worsening of distress. But oxazepam itself is a benzodiazepine, which may have an effect on tinnitus.^55^ Zöger et al.^52^ reported 24% attrition in this study, but none of the dropouts had received oxazepam, suggesting that it may have affected their result.

In summary, three RCTS examining antidepressants report three different findings. Meta-analysis was not performed.

### Anxiolytics

One RCT examined the efficacy of an anxiolytic. Jalali et al.^56^ compared alprazolam (a benzodiazepine) with chlorpheniramine (antihistamine), excluding participants scoring more than 14 points on the HAM-A anxiety questionnaire. They found no significant change in THI score between groups. Changes in anxiety and depression were not reported.

### Night Sedation

Two RCTs were identified that examined the efficacy of the neurohormone sedative melatonin. Neri et al.^57^ compared melatonin (3 mg at night for 80 nights) to a no-treatment control group. There were no significant changes in THI score between groups. Rosenberg et al.^58^ compared melatonin (3 mg at night for 30 nights) to placebo (lactose) and also found no significant effect on THI score between groups. Anxiety and depression were not measured in these studies. Meta-analysis was not performed.

## DISCUSSION

The aim of this review was to evaluate the evidence base for those tinnitus management interventions recommended by the Department of Health's Good Practice Guide.^5^ Where possible, we aimed to identify the conditions under which these interventions are efficacious, and consider whether these interventions are sufficiently researched or in need of further study. We also asked which patients benefit most, but report little on this question due to limited information in those studies reviewed.

In a previous review of RCTs of tinnitus interventions, Dobie did not find any that demonstrated a significant benefit of intervention over typically large placebo effects.^12^ He concluded that nowhere in the literature were there studies similar enough to attempt meta-analysis. Two recommendations from that review were that future RCTs needed consensus outcome measures and adequate sample sizes. There is a now reasonable consensus in the tinnitus research community on which validated measures to use.^59^ In this review, the most widely used tinnitus questionnaire was the TRQ. It appeared in 10 of the 28 studies and was used here in two separate meta-analyses. However, only seven of 28 studies used a power calculation to estimate their required sample size. Therefore, more than 10 years on, although we have greater RCT-level evidence and some opportunities for meta-analysis, there is still little evidence for the efficacy of most recommended treatment strategies.

It is striking that many authors ascribed null results for changes in depression and anxiety to the fact that participants have low baseline scores, often quoting floor effects. It may well be that, as authors suggest, more distressed individuals are less likely to put themselves forward for clinical trials. However, it may equally be that major depression or other comorbidities are less common than anecdotally suggested. Studies of the prevalence of comorbid depression or anxiety suggest it is no more an issues for tinnitus patients than it is for non-tinnitus ENT patients.^60^, ^61^ Given that the screening and management of anxiety and depression is highlighted in the GPG, more routine clinical assessment of anxiety and depression at baseline and outcome is needed to explore this observation.

### Improving the Quality of Tinnitus Research

Of the 28 studies identified, six were rated as low-level evidence, 17 as moderate, and five as high-level evidence (Table I). Unsurprisingly, four of the high-level trials involved drug therapies. Only one high-level trial of CBT was identified. For therapies involving sound enrichment or TRT, studies were rated as providing little or no evidence above low-level.

The main factor limiting study quality was blinding of participants to the intervention or expected outcome, and blinding of investigators to the expected outcome. There are ethical limitations to blinding that may be insurmountable; it would be unethical to deliver a psychological intervention such as CBT in a way that is wrong. Blinding of investigators, however, could be used more routinely as outcomes can easily be measured by a researcher who is blind to the intervention received. Only six of 28 studies reported the use of blinding, five of which were drug trials.

Use of appropriate controls is an issue. Given the nature of some tinnitus management interventions, a three-armed controlled design seems most appropriate (i.e., a formal intervention such as CBT or TRT compared to a simpler intervention such as a self-help book) and a no-intervention or waiting-list control. This design was used in some studies reviewed here and partly controls for placebo/nonspecific effects (e.g., learning more about tinnitus, clinical contact time) and toward identifying which components of the treatments are likely responsible for change.

Adequate numbers of participants are also important. Only seven of 28 RCTs conducted a power calculation of their required sample size, and only five met that sample size. Power calculations represent a simple way to improve future study quality in tinnitus research.

By design, all RCTs reviewed here reported validated measures of tinnitus intrusiveness, anxiety, or depression. Although many authors reported clinically as well as statistically significant changes, some limited their reports to the statistical changes only, which may have no real meaning in the clinic. In similar terms, it is also important to note the significance of nonspecific effects. For example, Rief et al.^42^ reported a 5-point reduction in TQ score as a significant improvement compared to waiting-list controls. However, elsewhere in the literature a 5-point reduction in TQ score (from a comparable baseline) was observed between two repeated measures,^62^ suggesting that this reduction is within the expected variability or test-retest value of the measure. More thorough reporting of clinical and statistical significance, or the provision of individual scores, is recommended for transparency and to facilitate future meta-analyses.

## CONCLUSION

Tinnitus care and research has long been and continues to be a challenge. The efficacy of most tinnitus management interventions recommended for clinical practice remains to be demonstrated conclusively as there are currently too few studies to make informed conclusions. Most studies reviewed here provide only moderate levels of evidence largely due to a lack of power and incomplete data reporting. Interventions most in need of high-level evidence studies are hearing aids/sound enrichment and TRT, whereas the efficacy of therapist-delivered CBT appears to be reasonably established. If audiologists are to deliver CBT, however, this will require new investigation. Although the overall quality of CBT studies is moderate, there is sufficient consistency within these results to be confident of its benefit for tinnitus intrusiveness. In terms of pharmacotherapy, only antidepressant use shows any evidence of potential benefit.
